# *cis*-2,6-Dibenzyl­cyclo­hexa­none

**DOI:** 10.1107/S1600536809004887

**Published:** 2009-02-21

**Authors:** John P. Culver, Sean Parkin, Peter A. Crooks

**Affiliations:** a Department of Pharmaceutical Sciences, College of Pharmacy, University of Kentucky, Lexington, KY 40536, USA; b Department of Chemistry, University of Kentucky, Lexington, KY 40506, USA

## Abstract

In the title compound, C_20_H_22_O, the mol­ecule is a *meso* isomer with the two benzyl groups *cis* to each other. The central cyclo­hexa­none ring adopts a chair conformation. The mol­ecule lies on a noncrystallographic mirror plane and the dihedral angles of the benzyl groups with respect to the ketone moiety are 88.06 (6) and 89.07 (6)°.

## Related literature

For background literature, see: Irvine *et al.* (1972 ▶); Corey *et al.* (1955 ▶); Ram & Ehrenkaufer (1988 ▶); Paryzek *et al.* (2003 ▶).
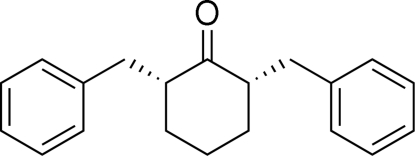


## Experimental

### 

#### Crystal data

C_20_H_22_O
*M* *_r_* = 278.38
Monoclinic, 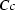

*a* = 30.1194 (12) Å
*b* = 5.5650 (2) Å
*c* = 9.3048 (5) Å
β = 98.276 (2)°
*V* = 1543.38 (12) Å^3^
*Z* = 4
Mo *K*α radiation
μ = 0.07 mm^−1^
*T* = 90 K
0.20 × 0.10 × 0.05 mm

#### Data collection

Nonius KappaCCD diffractometer
Absorption correction: multi-scan (*SCALEPACK*; Otwinowski & Minor, 1997 ▶) *T*_min_ = 0.986, *T*_max_ = 0.996
12091 measured reflections
1750 independent reflections
1574 reflections with *I* > 2σ(*I*)
*R*_int_ = 0.047

#### Refinement

*R*[*F* ^2^ > 2σ(*F* ^2^)] = 0.034
*wR*(*F* ^2^) = 0.081
*S* = 1.08
1750 reflections
191 parameters
2 restraints
H-atom parameters constrained
Δρ_max_ = 0.18 e Å^−3^
Δρ_min_ = −0.15 e Å^−3^

### 

Data collection: *COLLECT* (Nonius, 1999 ▶); cell refinement: *SCALEPACK* (Otwinowski & Minor, 1997 ▶); data reduction: *DENZO-SMN* (Otwinowski & Minor, 1997 ▶); program(s) used to solve structure: *SHELXS97* (Sheldrick, 2008 ▶); program(s) used to refine structure: *SHELXL97* (Sheldrick, 2008 ▶); molecular graphics: *XP* in *SHELXTL* (Sheldrick, 2008 ▶); software used to prepare material for publication: *SHELXL97* and local procedures.

## Supplementary Material

Crystal structure: contains datablocks global, I. DOI: 10.1107/S1600536809004887/pv2137sup1.cif

Structure factors: contains datablocks I. DOI: 10.1107/S1600536809004887/pv2137Isup2.hkl

Additional supplementary materials:  crystallographic information; 3D view; checkCIF report

## Figures and Tables

**Fig. 1.**
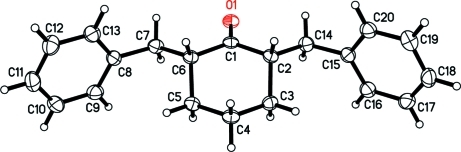
A view of the molecule with the atom numbering scheme. Displacement ellipsoids are drawn at the 50% probability level.

## supplementary crystallographic information

### Comment

The stereochemistry of α,α'-dibenzylcycloalkanones (Irvine *et al.* 1972) and 2,6-dibenzylcyclohexanones (Corey *et al.* 1955), ammonium formate in organic synthesis: a versatile agent in catalytic hydrogen transfer reductions (Ram & Ehrenkaufer, 1988), and ammonium formate/palladium on carbon: A versatile system for catalytic hydrogen transfer reductions of carbon–carbon double bonds (Paryzek *et al.* 2003), are a few of the articles related to the study of our research. In the investigation of possible treatments for the abuse of methamphetamine, we have undertaken the design, synthesis, and structural analysis of a series of (*cis*)-2,6-dibenzylcyclohexan-1-one analogs and derivatives with variable substituents on the benzene ring. The primary goal of the X-ray analysis of the title compound was to confirm the *cis* stereochemistry of the benzyl substituents at C2 and C6 of the cyclohexanone ring, and to obtain detailed information on the structural conformation of the molecule for use in structure–activity relationship (SAR) studies.

The molecular structure of the title compound (Fig. 1) established the *cis* stereochemistry of the C2 and C6 benzyl substituents. The central cyclohexanone ring has a chair conformation. The molecule lies on a non-crystallographic mirror plane which passes through atoms O1, C1 and C4 and hence the torsion angles C1—C2—C14—C15 and C1—C6—C7—C8 are very similar (176.75 (16) and -176.89 (17)°, respectively). The dihedral angles of the benzyl groups with respect to the ketone moiety are 88.06 (6) and 89.07 (6)°.

### Experimental

A mixture of cyclohexanone (1.0 g, 10.2 mmol), benzaldehyde (2.3 g, 21.7 mmol), and potassium hydroxide (1.22 g, 21.7 mmol) was stirred in methanol (20 ml) at ambient temperature for 4 h. The yellow solid precipitate was collected by filtration, and washed with cold methanol to yield the crude 2,6-dibenzylidenecyclohexanone (2.6 g, 9.5 mmol). A portion of the 2,6-dibenzylidenecyclohexanone product (1.0 g, 3.65 mmol) was subjected to hydrogenation *via* addition of palladium (10% on carbon, 0.1 g) and an excess of ammonium formate (2.4 g, 38.1 mmol) and then brought to reflux in methanol (50 ml) for 4 h. After cooling to ambient temperature, the reaction mixture was filtered, and the solvent was evaporated under vacuum. Chloroform (5 ml) was added to precipitate the remaining excess of ammonium formate, which was then removed by filtration. The residue was subjected to flash chromatography (solvent system 50:1 hexane–ethyl acetate). The crude product was then evaporated to dryness and crystallized from methanol to yield (*cis*)-2,6-dibenzylcyclohexanone (0.48 g, 1.72 mmol) as a colorless crystalline solid, that was suitable for X-ray analysis.

### Refinement

All H atoms were located in difference Fourier syntheses, and refined using riding models with bond distances of 0.95 Å (C_ar_—H), 0.99 Å (C_sec_—H), and 1.00 Å (C*_tert_*—H). Isotropic H-atom displacement parameters were set to 1.2*U*_eq_ of the parent atom. Friedel opposites were merged for this structure because of the absence of any anomalous scattering with which to refine a physically meaningful value of the Flack parameter.

### Crystal data

**Table:**

C_20_H_22_O	*F*(000) = 600
*M**_r_* = 278.38	*D*_x_ = 1.198 Mg m^−^^3^
Monoclinic, *C**c*	Mo *K*α radiation, λ = 0.71073 Å
Hall symbol: C -2yc	Cell parameters from 1926 reflections
*a* = 30.1194 (12) Å	θ = 1.0–27.5°
*b* = 5.5650 (2) Å	µ = 0.07 mm^−^^1^
*c* = 9.3048 (5) Å	*T* = 90 K
β = 98.276 (2)°	Block cut from needle, colourless
*V* = 1543.38 (12) Å^3^	0.20 × 0.10 × 0.05 mm
*Z* = 4	

### Data collection

**Table:**

Nonius KappaCCD diffractometer	1750 independent reflections
Radiation source: fine-focus sealed tube	1574 reflections with *I* > 2σ(*I*)
graphite	*R*_int_ = 0.047
Detector resolution: 18 pixels mm^-1^	θ_max_ = 27.5°, θ_min_ = 1.4°
ω scans at fixed χ = 55°	*h* = −37→38
Absorption correction: multi-scan (*SCALEPACK*; Otwinowski & Minor, 1997)	*k* = −6→7
*T*_min_ = 0.986, *T*_max_ = 0.996	*l* = −12→11
12091 measured reflections	

### Refinement

**Table:**

Refinement on *F*^2^	Secondary atom site location: difference Fourier map
Least-squares matrix: full	Hydrogen site location: inferred from neighbouring sites
*R*[*F*^2^ > 2σ(*F*^2^)] = 0.034	H-atom parameters constrained
*wR*(*F*^2^) = 0.081	*w* = 1/[σ^2^(*F*_o_^2^) + (0.0379*P*)^2^ + 0.4656*P*] where *P* = (*F*_o_^2^ + 2*F*_c_^2^)/3
*S* = 1.08	(Δ/σ)_max_ = 0.001
1750 reflections	Δρ_max_ = 0.18 e Å^−^^3^
191 parameters	Δρ_min_ = −0.14 e Å^−^^3^
2 restraints	Extinction correction: *SHELXL97* (Sheldrick, 2008), Fc^*^=kFc[1+0.001xFc^2^λ^3^/sin(2θ)]^-1/4^
Primary atom site location: structure-invariant direct methods	Extinction coefficient: 0.013 (2)

### Special details

**Table:**

Experimental. ^1^H NMR (CDCl_3_): δ 1.364 (*dt*, 2H), 1.47–1.63 (*m*, 1H), 1.73–1.82 (*m*, 1H), 2.01–2.09 (*m*, 2H), 2.424 (*dd*, 2H), 2.52–2.63 (*m*, 2H), 3.235 (*dd*, 2H), 7.13–7.30 (*m*, 10H); ^13^C NMR (CDCl_3_): δ 25.65 (C4), 35.15 (C3,C5), 35.78 (C7,C14), 53.15 (C2,C6), 126.04 (C11,C18), 128.39 (C9, C13, C16, C20), 129.28 (C10, C12, C17, C19), 140.68 (C8, C15), 212.80 (C1). m.p. 393–395 K (lit. m.p. 395 K)
Geometry. All e.s.d.'s (except the e.s.d. in the dihedral angle between two l.s. planes) are estimated using the full covariance matrix. The cell e.s.d.'s are taken into account individually in the estimation of e.s.d.'s in distances, angles and torsion angles; correlations between e.s.d.'s in cell parameters are only used when they are defined by crystal symmetry. An approximate (isotropic) treatment of cell e.s.d.'s is used for estimating e.s.d.'s involving l.s. planes.
Refinement. Refinement of *F*^2^ against ALL reflections. The weighted *R*-factor *wR* and goodness of fit *S* are based on *F*^2^, conventional *R*-factors *R* are based on *F*, with *F* set to zero for negative *F*^2^. The threshold expression of *F*^2^ > 2σ(*F*^2^) is used only for calculating *R*-factors(gt) *etc*. and is not relevant to the choice of reflections for refinement. *R*-factors based on *F*^2^ are statistically about twice as large as those based on *F*, and *R*- factors based on ALL data will be even larger.

### Fractional atomic coordinates and isotropic or equivalent isotropic displacement parameters (Å^2^)

**Table:**

	*x*	*y*	*z*	*U*_iso_*/*U*_eq_	
O1	0.37985 (5)	−0.0297 (3)	0.40486 (14)	0.0253 (3)	
C1	0.38084 (7)	0.1057 (3)	0.50806 (19)	0.0208 (4)	
C2	0.33796 (7)	0.1967 (4)	0.5576 (2)	0.0220 (4)	
H2	0.3349	0.1141	0.6512	0.026*	
C3	0.34065 (7)	0.4687 (4)	0.5882 (2)	0.0241 (4)	
H3A	0.3397	0.5567	0.4954	0.029*	
H3B	0.3143	0.5186	0.6335	0.029*	
C4	0.38336 (7)	0.5351 (4)	0.6884 (2)	0.0242 (4)	
H4A	0.3841	0.7109	0.7049	0.029*	
H4B	0.3835	0.4550	0.7835	0.029*	
C5	0.42475 (7)	0.4597 (4)	0.6235 (2)	0.0235 (4)	
H5A	0.4520	0.5033	0.6913	0.028*	
H5B	0.4256	0.5481	0.5315	0.028*	
C6	0.42510 (7)	0.1872 (4)	0.5934 (2)	0.0224 (4)	
H6	0.4286	0.1033	0.6894	0.027*	
C7	0.46483 (7)	0.1121 (4)	0.5165 (2)	0.0257 (5)	
H7A	0.4636	−0.0638	0.5006	0.031*	
H7B	0.4618	0.1907	0.4201	0.031*	
C8	0.51001 (7)	0.1770 (4)	0.6010 (2)	0.0252 (4)	
C9	0.53381 (7)	0.3759 (4)	0.5643 (2)	0.0307 (5)	
H9	0.5220	0.4712	0.4830	0.037*	
C10	0.57469 (8)	0.4375 (4)	0.6453 (3)	0.0354 (5)	
H10	0.5906	0.5742	0.6192	0.042*	
C11	0.59215 (7)	0.2998 (5)	0.7638 (3)	0.0363 (6)	
H11	0.6200	0.3419	0.8194	0.044*	
C12	0.56876 (8)	0.1000 (4)	0.8012 (3)	0.0343 (5)	
H12	0.5805	0.0052	0.8828	0.041*	
C13	0.52826 (7)	0.0393 (4)	0.7196 (2)	0.0300 (5)	
H13	0.5126	−0.0990	0.7450	0.036*	
C14	0.29661 (7)	0.1293 (4)	0.4477 (2)	0.0260 (4)	
H14A	0.2990	0.2070	0.3534	0.031*	
H14B	0.2963	−0.0468	0.4324	0.031*	
C15	0.25298 (7)	0.2043 (4)	0.4972 (2)	0.0255 (4)	
C16	0.23059 (7)	0.4124 (4)	0.4440 (2)	0.0306 (5)	
H16	0.2420	0.5051	0.3718	0.037*	
C17	0.19167 (8)	0.4852 (4)	0.4958 (3)	0.0345 (5)	
H17	0.1767	0.6277	0.4592	0.041*	
C18	0.17461 (7)	0.3511 (4)	0.6005 (3)	0.0346 (5)	
H18	0.1483	0.4028	0.6369	0.042*	
C19	0.19597 (7)	0.1412 (4)	0.6521 (2)	0.0344 (5)	
H19	0.1840	0.0469	0.7225	0.041*	
C20	0.23492 (7)	0.0694 (4)	0.6003 (2)	0.0294 (5)	
H20	0.2495	−0.0745	0.6360	0.035*	

### Atomic displacement parameters (Å^2^)

**Table:**

	*U*^11^	*U*^22^	*U*^33^	*U*^12^	*U*^13^	*U*^23^
O1	0.0313 (7)	0.0223 (7)	0.0228 (7)	−0.0010 (6)	0.0052 (6)	−0.0033 (6)
C1	0.0280 (9)	0.0163 (9)	0.0187 (9)	−0.0008 (8)	0.0059 (7)	0.0043 (7)
C2	0.0248 (9)	0.0209 (10)	0.0207 (9)	−0.0020 (8)	0.0041 (7)	−0.0017 (8)
C3	0.0268 (10)	0.0224 (10)	0.0235 (10)	0.0015 (8)	0.0056 (8)	−0.0015 (8)
C4	0.0305 (10)	0.0212 (10)	0.0213 (10)	−0.0008 (8)	0.0042 (8)	−0.0017 (8)
C5	0.0266 (10)	0.0222 (10)	0.0221 (9)	−0.0019 (8)	0.0048 (8)	−0.0012 (8)
C6	0.0249 (9)	0.0210 (10)	0.0220 (9)	−0.0011 (8)	0.0059 (7)	0.0004 (7)
C7	0.0283 (10)	0.0243 (11)	0.0263 (10)	−0.0011 (8)	0.0095 (8)	−0.0028 (8)
C8	0.0227 (10)	0.0248 (11)	0.0298 (10)	−0.0009 (8)	0.0093 (8)	−0.0052 (8)
C9	0.0315 (11)	0.0255 (11)	0.0362 (12)	0.0001 (9)	0.0080 (9)	0.0040 (9)
C10	0.0295 (11)	0.0307 (13)	0.0471 (14)	−0.0058 (10)	0.0093 (10)	0.0023 (10)
C11	0.0248 (11)	0.0434 (15)	0.0409 (13)	−0.0023 (10)	0.0057 (9)	−0.0023 (11)
C12	0.0287 (11)	0.0402 (13)	0.0348 (12)	0.0030 (10)	0.0068 (9)	0.0053 (10)
C13	0.0268 (10)	0.0296 (12)	0.0356 (12)	−0.0008 (9)	0.0114 (9)	0.0042 (10)
C14	0.0262 (10)	0.0267 (11)	0.0242 (10)	−0.0008 (8)	0.0003 (8)	−0.0015 (8)
C15	0.0236 (10)	0.0249 (11)	0.0268 (10)	−0.0014 (9)	−0.0006 (8)	−0.0034 (9)
C16	0.0280 (10)	0.0286 (12)	0.0343 (11)	−0.0015 (9)	0.0011 (9)	0.0019 (9)
C17	0.0282 (11)	0.0277 (12)	0.0454 (14)	0.0010 (9)	−0.0024 (10)	−0.0034 (10)
C18	0.0231 (10)	0.0389 (13)	0.0412 (12)	−0.0023 (10)	0.0026 (9)	−0.0107 (11)
C19	0.0298 (11)	0.0407 (13)	0.0328 (11)	−0.0069 (10)	0.0049 (9)	−0.0011 (10)
C20	0.0275 (11)	0.0275 (11)	0.0315 (11)	−0.0024 (9)	−0.0007 (9)	0.0008 (9)

### Geometric parameters (Å, °)

**Table:**

O1—C1	1.217 (2)	C9—H9	0.9500
C1—C2	1.519 (3)	C10—C11	1.383 (3)
C1—C6	1.520 (3)	C10—H10	0.9500
C2—C14	1.540 (3)	C11—C12	1.387 (3)
C2—C3	1.541 (3)	C11—H11	0.9500
C2—H2	1.0000	C12—C13	1.383 (3)
C3—C4	1.521 (3)	C12—H12	0.9500
C3—H3A	0.9900	C13—H13	0.9500
C3—H3B	0.9900	C14—C15	1.513 (3)
C4—C5	1.520 (3)	C14—H14A	0.9900
C4—H4A	0.9900	C14—H14B	0.9900
C4—H4B	0.9900	C15—C20	1.389 (3)
C5—C6	1.542 (3)	C15—C16	1.394 (3)
C5—H5A	0.9900	C16—C17	1.391 (3)
C5—H5B	0.9900	C16—H16	0.9500
C6—C7	1.538 (3)	C17—C18	1.383 (4)
C6—H6	1.0000	C17—H17	0.9500
C7—C8	1.514 (3)	C18—C19	1.386 (3)
C7—H7A	0.9900	C18—H18	0.9500
C7—H7B	0.9900	C19—C20	1.390 (3)
C8—C9	1.388 (3)	C19—H19	0.9500
C8—C13	1.390 (3)	C20—H20	0.9500
C9—C10	1.391 (3)		
			
O1—C1—C2	121.35 (18)	C13—C8—C7	120.24 (19)
O1—C1—C6	121.12 (17)	C8—C9—C10	120.8 (2)
C2—C1—C6	117.52 (16)	C8—C9—H9	119.6
C1—C2—C14	111.03 (16)	C10—C9—H9	119.6
C1—C2—C3	111.05 (16)	C11—C10—C9	120.1 (2)
C14—C2—C3	112.23 (17)	C11—C10—H10	120.0
C1—C2—H2	107.4	C9—C10—H10	120.0
C14—C2—H2	107.4	C10—C11—C12	119.7 (2)
C3—C2—H2	107.4	C10—C11—H11	120.1
C4—C3—C2	111.66 (17)	C12—C11—H11	120.1
C4—C3—H3A	109.3	C13—C12—C11	119.8 (2)
C2—C3—H3A	109.3	C13—C12—H12	120.1
C4—C3—H3B	109.3	C11—C12—H12	120.1
C2—C3—H3B	109.3	C12—C13—C8	121.2 (2)
H3A—C3—H3B	107.9	C12—C13—H13	119.4
C5—C4—C3	111.04 (15)	C8—C13—H13	119.4
C5—C4—H4A	109.4	C15—C14—C2	112.67 (16)
C3—C4—H4A	109.4	C15—C14—H14A	109.1
C5—C4—H4B	109.4	C2—C14—H14A	109.1
C3—C4—H4B	109.4	C15—C14—H14B	109.1
H4A—C4—H4B	108.0	C2—C14—H14B	109.1
C4—C5—C6	111.74 (16)	H14A—C14—H14B	107.8
C4—C5—H5A	109.3	C20—C15—C16	118.5 (2)
C6—C5—H5A	109.3	C20—C15—C14	120.31 (19)
C4—C5—H5B	109.3	C16—C15—C14	121.21 (19)
C6—C5—H5B	109.3	C17—C16—C15	120.5 (2)
H5A—C5—H5B	107.9	C17—C16—H16	119.7
C1—C6—C7	111.01 (16)	C15—C16—H16	119.7
C1—C6—C5	111.09 (17)	C18—C17—C16	120.3 (2)
C7—C6—C5	112.18 (16)	C18—C17—H17	119.9
C1—C6—H6	107.4	C16—C17—H17	119.9
C7—C6—H6	107.4	C17—C18—C19	119.9 (2)
C5—C6—H6	107.4	C17—C18—H18	120.1
C8—C7—C6	113.27 (16)	C19—C18—H18	120.1
C8—C7—H7A	108.9	C18—C19—C20	119.7 (2)
C6—C7—H7A	108.9	C18—C19—H19	120.2
C8—C7—H7B	108.9	C20—C19—H19	120.2
C6—C7—H7B	108.9	C15—C20—C19	121.2 (2)
H7A—C7—H7B	107.7	C15—C20—H20	119.4
C9—C8—C13	118.4 (2)	C19—C20—H20	119.4
C9—C8—C7	121.35 (19)		
			
O1—C1—C2—C14	−9.2 (3)	C7—C8—C9—C10	178.18 (19)
C6—C1—C2—C14	171.62 (17)	C8—C9—C10—C11	0.1 (3)
O1—C1—C2—C3	−134.82 (18)	C9—C10—C11—C12	0.2 (4)
C6—C1—C2—C3	46.0 (2)	C10—C11—C12—C13	0.2 (4)
C1—C2—C3—C4	−50.9 (2)	C11—C12—C13—C8	−0.9 (3)
C14—C2—C3—C4	−175.87 (16)	C9—C8—C13—C12	1.1 (3)
C2—C3—C4—C5	58.5 (2)	C7—C8—C13—C12	−177.8 (2)
C3—C4—C5—C6	−58.3 (2)	C1—C2—C14—C15	176.75 (16)
O1—C1—C6—C7	9.5 (3)	C3—C2—C14—C15	−58.3 (2)
C2—C1—C6—C7	−171.33 (17)	C2—C14—C15—C20	−78.3 (2)
O1—C1—C6—C5	135.05 (18)	C2—C14—C15—C16	100.0 (2)
C2—C1—C6—C5	−45.8 (2)	C20—C15—C16—C17	1.5 (3)
C4—C5—C6—C1	50.5 (2)	C14—C15—C16—C17	−176.9 (2)
C4—C5—C6—C7	175.41 (16)	C15—C16—C17—C18	−0.3 (3)
C1—C6—C7—C8	−176.89 (17)	C16—C17—C18—C19	−1.1 (3)
C5—C6—C7—C8	58.2 (2)	C17—C18—C19—C20	1.2 (3)
C6—C7—C8—C9	−101.5 (2)	C16—C15—C20—C19	−1.3 (3)
C6—C7—C8—C13	77.4 (2)	C14—C15—C20—C19	177.04 (19)
C13—C8—C9—C10	−0.7 (3)	C18—C19—C20—C15	0.0 (3)
